# Quantitative autistic trait measurements index background genetic risk for ASD in Hispanic families

**DOI:** 10.1186/s13229-016-0100-1

**Published:** 2016-09-06

**Authors:** Joshua Page, John Nicholas Constantino, Katherine Zambrana, Eden Martin, Ilker Tunc, Yi Zhang, Anna Abbacchi, Daniel Messinger

**Affiliations:** 1 Department of Psychiatry and Pediatrics, Washington University School of Medicine, 4444 Forest Park Ave, St. Louis, MO USA; 2 Department of Psychology, University of Miami, P.O. Box 248185-0751, Coral Gables, FL USA; 3 The Center for Genetic Epidemiology and Statistical Genetics, University of Miami School of Medicine, 1501 NW 10th Avenue, Miami, FL USA; 4 Division of Intramural Research, National Heart Lung and Blood Institute, NIH, 31 Center Dr., Bethesda, MD USA

**Keywords:** Measurement, Social Responsiveness Scale, Hispanic, Ancestry, Assortative mating

## Abstract

**Background:**

Recent studies have indicated that quantitative autistic traits (QATs) of parents reflect inherited liabilities that may index background genetic risk for clinical autism spectrum disorder (ASD) in their offspring. Moreover, preferential mating for QATs has been observed as a potential factor in concentrating autistic liabilities in some families across generations. Heretofore, intergenerational studies of QATs have focused almost exclusively on Caucasian populations—the present study explored these phenomena in a well-characterized Hispanic population.

**Methods:**

The present study examined QAT scores in siblings and parents of 83 Hispanic probands meeting research diagnostic criteria for ASD, and 64 non-ASD controls, using the Social Responsiveness Scale-2 (SRS-2). Ancestry of the probands was characterized by genotype, using information from 541,929 single nucleotide polymorphic markers.

**Results:**

In families of Hispanic children with an ASD diagnosis, the pattern of quantitative trait correlations observed between ASD-affected children and their first-degree relatives (ICCs on the order of 0.20), between unaffected first-degree relatives in ASD-affected families (sibling/mother ICC = 0.36; sibling/father ICC = 0.53), and between spouses (mother/father ICC = 0.48) were in keeping with the influence of transmitted background genetic risk and strong preferential mating for variation in quantitative autistic trait burden. Results from analysis of ancestry-informative genetic markers among probands in this sample were consistent with that from other Hispanic populations.

**Conclusions:**

Quantitative autistic traits represent measurable indices of inherited liability to ASD in Hispanic families. The accumulation of autistic traits occurs within generations, between spouses, and across generations, among Hispanic families affected by ASD. The occurrence of preferential mating for QATs—the magnitude of which may vary across cultures—constitutes a mechanism by which background genetic liability for ASD can accumulate in a given family in successive generations.

## Background

Recent studies have indicated that a substantial proportion of inherited liability to autism spectrum disorders (ASD) can be attributed to common, inherited variants [1–4]. Even in patients carrying specific deleterious ASD-associated mutations (e.g., the 16p11.2 deletion), significant variation in phenotypic expression is associated with variations in social responsiveness, intelligence, and other phenotypic markers among first-degree relatives [5].

These associations underscore the role of background genetic factors in risk for ASD. Quantitative trait scales such as the Social Responsiveness Scale-2 (SRS-2) [6], and the Broader Autism Phenotype Questionnaire [7] enable the identification of subclinical autistic traits in relatives of ASD individuals. Significant associations of SRS-2 scores in twins and siblings have established the influence of genetic factors within a given generation [8, 9]. Similarly, significant associations between parents and their offspring for SRS-2 scores demonstrate the transmission of autistic traits *between* generations through heritable genetic factors.

Previous studies have also demonstrated evidence of assortative mating for sociality through spousal correlations in social responsiveness. Three previous studies involving both ASD-affected and ASD-unaffected families have reported significant spousal correlation for SRS-2 scores ranging from 0.26 to 0.39 [9–11]. Preferential mate selection between individuals with similar degrees of social responsiveness can conceivably lead to an aggregation of QAT in successive generations of a family. Lyall et al. [9] demonstrated that when both members of a spousal pair fell within the upper quintile of the population distribution for SRS-2 scores, it doubled the relative risk of an offspring carrying a clinical-ASD diagnosis [9].

Studies of preferential mating for QAT have predominantly examined Caucasian populations, and it is possible that this phenomenon varies across cultures. Given the implications for accumulated liability in successive generations, and the fact that the influence of specific inherited liabilities for many complex genetic conditions have been shown to differ by ancestral origin [12–15], cross-cultural research in family genetic influences on disease is of substantial importance. Particularly, under-representation of minority families (as characterizes much of the current ASD genetic research literature) [16] sets up the scenario of missing variants that may exhibit enhanced effects against specific ancestral backgrounds or generating assumptions about causation that relate exclusively to a majority race. The primary aim of the present study is to characterize the aggregation of quantitative autistic traits in family members of Hispanic ASD-diagnosed individuals, among whom ancestral origin was specified by genotype, using methods for quantitative trait analysis that have been implemented in previous studies involving predominantly Caucasian family samples [10].

## Methods

### Study population

The study population was comprised of 151 Hispanic families recruited from the University of Miami Department of Psychology (UM), categorized into two groups by index subject: ASD diagnosis (*n* = 85) and typical controls (*n* = 66). ASD diagnoses were ascertained from UM affiliated child/adolescent psychiatric clinics. The typical controls were families recruited from South Florida, including the University of Miami student community. All families contained at least one English-speaking parent, one parent of self-reported Hispanic ancestry, and one sibling. In 79.6 % of families, both parents were Hispanic. Five of the ASD families were known multiple-incidence autism families. One affected sibling in the family was designated as the proband/subject case to maintain independence.

In our present study, there were 604 individuals. We collected 480 English-translation Social Responsiveness Scale-2 (SRS-2) and 83 Spanish-translation SRS-2 forms (data unavailable for 41 individuals). Three different forms were administered based on age of the subject (25 preschool, 177 school-aged, 350 adult; data unavailable or preverbal subject for 52 individuals). We also differentiated the sample by informant (285 parent/caretaker, 265 spouse, data unavailable for 54 individuals). SRS-2 scores were obtained from student’s teachers for 50 subject cases (45 w/ASD diagnoses) and 43 of their siblings. Eleven preverbal individuals were evaluated with a video-referenced rating of reciprocal social behavior [17] and these were excluded from all analyses of SRS-2 data. All ASD subjects were diagnosed by expert clinicians at the University of Miami site; in addition to the SRS-2, either the Autism Diagnostic Interview-Revised (ADI-R) or the Social Communication Questionnaire (SCQ) provided diagnostic confirmation by developmental history.

### Measures

The Social Responsiveness Scale-2 (SRS-2) is an extensively validated [6, 18] quantitative measure of characterizing traits and symptoms of the autistic syndrome. The SRS-2 has been shown to have high sensitivity to detect milder degrees of social impairment, and high specificity to differentiate ASD from other conditions. The instrument is normed by sex and informant type.

Although IQ data were not available for the sample, previous research has demonstrated that SRS-2 scores differ minimally as a function of IQ among verbal subjects [6, 19, 20]. In this sample, verbal ASD subjects had a mean SRS-2 score of 98.6 (*N* = 61, SD = 26.6); non-verbal ASD subjects had a mean SRS-2 score of 104.8 (*N* = 21, SD = 34.1)—this difference did not reach statistical significance (*p* = 0.20).

Fombonne et al. (2012) recently published findings on the psychometric properties of the Spanish version of the SRS-2 from a case-control study of ASD in Mexico [21]. In this sample of 563 children (*N* = 200 ASD group*, N* = 363 typically developing group; matched for age and gender), the SRS-2 exhibited high discriminant validity between ASD and typical controls for both total score and subscale scores. The ASD sample mean for SRS-2 total raw score (102.2) was in keeping with means reported in previous US studies involving exclusively English-speaking informants [6].

On average, respondents completing the Spanish version of the scale in this sample reported slightly higher SRS-2 scores than those completing the English version. This difference reached statistical significance difference for ASD-diagnosed children and their parents but not for siblings of ASD probands or for non-ASD families (Table 1).

**Table 1:** Comparison of English and Spanish SRS-2 Data in families of ASD subjects. Typical (control) families are not included due to insufficient sample size for Spanish language SRS-2

N.B. columns represent independent groups of subjects	English version SRS-2 mean (SD)	Spanish version SRS-2 mean (SD)	*t* test
ASD-diagnosed children	95.2 (27.8) *N* = 64	115.8 (26.9) *N* = 19	*t* = −2.924, df = 85, *p* < .004
Sibs of ASD cases	27.3 (20.6) *N* = 53	37.6 (25.3) *N* = 20	*t* = −1.751, df = 67, *p* < .084
Mothers of ASD subjects	29.6 (20.1) *N* = 47	50.1 (33.2) *N* = 17	*t* = −2.397, df = 20, *p* < .026
Fathers of ASD subjects	41.4 (33.2) *N* = 58	60.7 (34.1) *N* = 18	*t* = −2.132, df = 74, *p* < .036

### Genotyping and ancestral analysis

Sixty nine ASD probands with sufficient DNA quantity and quality were genotyped using Illumina HumanCoreExome Chips (Illumina, San Diego) according to standard genotyping protocols. A total of 542,584 SNPs were genotyped. Quality filtering was performed, checking for low efficiency samples and SNPs. 656 SNPs with a call rate <90 % were removed and all samples had a >98 % call rate. No duplicate samples were identified and all individuals were confirmed as unrelated. After quality control screening there were 541,929 SNPs in 69 individuals.

To contextualize the ancestry-informative genotypic data from the 69 ASD subjects, we included data from several publically available datasets: 165 CEU samples, 167 YRI samples, and 77 MEX samples were obtained from the HapMap project [22] representing European, African, and Mexican populations, respectively; 63 Native American samples from the Human Genome Diversity Project (HGDP) were also included. Additionally, we included 171 unrelated individuals from the Genetic Origins and Admixture of Latinos (GOAL) study: 53 Colombian, 58 Cuban, 24 Dominican, 4 Haitian, 14 Honduran, and 18 Puerto Rican individuals [23].

Principal component analysis (PCA) was used for genomic data in order to elucidate the patterns of population structure in the 69 ASD subjects. PCA was performed in EIGENSOFT [24] jointly for the 69 subjects from this study, samples from the GOAL study, HGDP, and HapMap reference populations. We selected the 39,035 SNPs that were in common among the samples in this study and the genotype data from the reference populations (out of 906,598 SNPs in GOAL study, 660,918 SNPs in HGDP Native American, and 1,440,616 in HapMap YRI, CEU, MEX). To perform global ancestry, we used the 135,388 SNPs in common among the study samples, HapMap CEU, YRI, and Native American populations. Ancestry proportions of each subject were determined as fractions relative to the European (CEU), Yoruba African (YRI), and HGDP Native American reference populations using ADMIXTURE [25]. For comparison of means by ancestral origin the group was divided in half by degree of CEU ancestry. Thirty four individuals had CEU ancestry >0.770 and 35 had CEU ancestry <0.770.

### Phenotypic data analysis

We analyzed SRS-2 distributions independently for ASD-diagnosed subjects, non-ASD controls, their siblings, and biological parents in order to characterize variations in distributions of SRS-2 scores by diagnostic status, language of the test, and informant. Intraclass correlations were calculated for associations between first-degree relatives separately by diagnostic group—ASD families and non-ASD families. For the children, the use of parent-report SRS-2 data optimized statistical power. There were insufficient multiplex families (*n* = 5) for separate analysis.

**Fig. 1:**
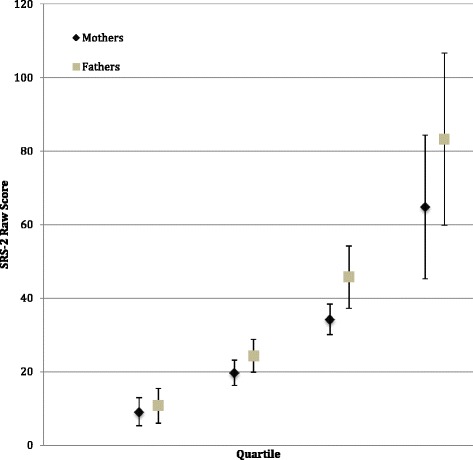
SRS-2 spouse-report data (raw) by quartile groups for mothers and fathers

Whenever appropriate, raw SRS-2 data were square-root-transformed to more closely approximate normality assumptions for the analyses (e.g., minimizing inaccuracies in the calculation of correlation coefficients that might be incurred by skewing of the SRS-2 distributions in the respective subject groupings). Finally, we examined whether concordant elevation in parental scores of ASD subjects occurred more frequently than (a) would be predicted by chance; and (b) would occur among parents of non-ASD subjects. We did so by dividing the raw spouse-report SRS-2 data into four quartiles based on parental severity of SRS-2 scores as presented in Fig. 1. All statistical analyses were implemented using SPSS v23.

## Results

### Quantitative autistic trait scores

**Fig. 2:**
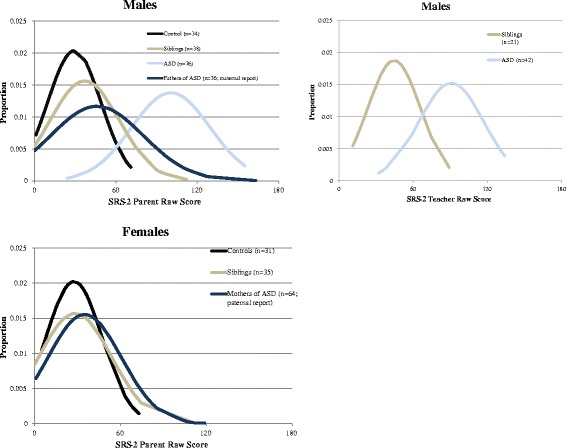
Normalized SRS-2 distributions by gender and parent/teacher-report. Due to insufficient sample size, data was excluded for ASD-diagnosed females, teacher-report data for male controls, and teacher-report data for female controls. Additionally, SRS-2 spousal report distributions were included for mothers and fathers of ASD children

**Fig. 3:**
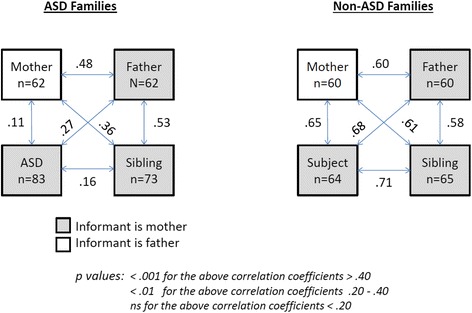
Intraclass coefficients of correlation for pairings of square-root-transformed SRS-2 scores of first-degree relatives

In keeping with prior studies in non-Hispanic samples [26], SRS-2 scores of children with ASD diagnosis were three standard deviations higher than those of typical controls (Table 2, Fig. 2), both by parent- and teacher-report. Whereas the intraclass correlation coefficient between ASD probands and their siblings was 0.16 (df = 79, *p* = 0.074; Fig. 1), typical siblings had an ICC of 0.71 (df = 65, *p* < 0.001; Fig. 3). As has been observed in prior studies, QATs aggregated in the male siblings of ASD probands, though to a lower degree than in previous-reports involving samples with higher proportions of multiplex families. The aggregation observed in this study was highly clinically and statistically significant when considering teacher-informant data, but did not reach statistical significance when exclusively incorporating parent data (this discrepancy has been observed in prior studies and may be related to subtle rater contrast effects for parent-report data in ASD-affected families [27]).

**Table 2:** Mean SRS-2 scores across groups for probands, siblings, and biological parents

Individual assessed	SRS-2 parent-report		SRS-2 teacher-report		Spouse-report
	Males	Females	Males	Females	
ASD-diagnosed subjects	100.5 (±28.9) *N* = 76	105.0 (±21.6) *N* = 7	90.7 (±26.2) *N* = 42	123.3 (±19.6) *N* = 3	–
Sibs of ASD cases	37.0 (±25.5) *N* = 38	28.4 (±25.4) *N* = 35	44.4 (±21.3) *N* = 21	40.0 (±27.8) *N* = 13	–
Typical control subjects	29.3 (±19.6) *N* = 34	27.6 (±19.7) *N* = 30	14.0 (±20.0) *N* = 3	32.5 (±14.9) *N* = 2	–
Siblings of typical controls	32.3 (±21.1) *N* = 48	35.2 (±22.3) *N* = 17	19.5 (±17.3) *N* = 8	27.0 (±4.2) *N* = 2	–
Mothers of ASD subjects	–	–	–	–	35.0 (±25.6) *N* = 64
Fathers of ASD subjects	–	–	–	–	46.0 (±34.2) *N* = 76
Mothers of typical controls	–	–	–	–	28.6 (±20.1) *N* = 62
Fathers of typical controls	–	–	–	–	34.1 (±23.3) *N* = 63

Trait correlations for specific pairings of first-degree relatives in both ASD and non-ASD families were generally moderate-to-strong, as shown in Fig. 3. The spouse-report SRS-2 intra class correlation coefficient between biological mothers and fathers of children with ASD was 0.48 (df = 61, *p* < .0001), and 0.60 for spousal pairs in non-ASD families (df = 59, *p* < .0001) (Fig. 3), both represent the highest reported spousal correlations for SRS-2 data to date. In this sample, concordant elevations of mothers and fathers (i.e., *both* parents in *upper* quartile of the sample distribution) were observed among ASD families than among non-ASD families (16.1 vs. 3.3 %, Fisher’s exact, *p* = .03), and the former substantially exceeded the proportion expected by chance, which was 6.25 %. These results were unchanged when restricting the analysis to data from one or the other specific language versions of the SRS-2 (Spanish vs. English).

Five out of 125 (4 %) parents in the control group and 22 out of 140 parents of ASD probands (15 %) had SRS *t* scores >70. Although diagnostic information about the higher scoring individuals (predominantly fathers) is not available, these distributions are in keeping with prior studies in both the general population and in clinical family studies [26]. Concern about potential biases inherent in ratings made by higher scoring adults in this sample is mitigated by the fact that paternal ratings of mothers’ SRS scores (Fig. 2) were entirely consistent with those observed in a large US epidemiologic sample, the Nurses Health II Study [9].

### Ancestry analysis

**Fig. 4:**
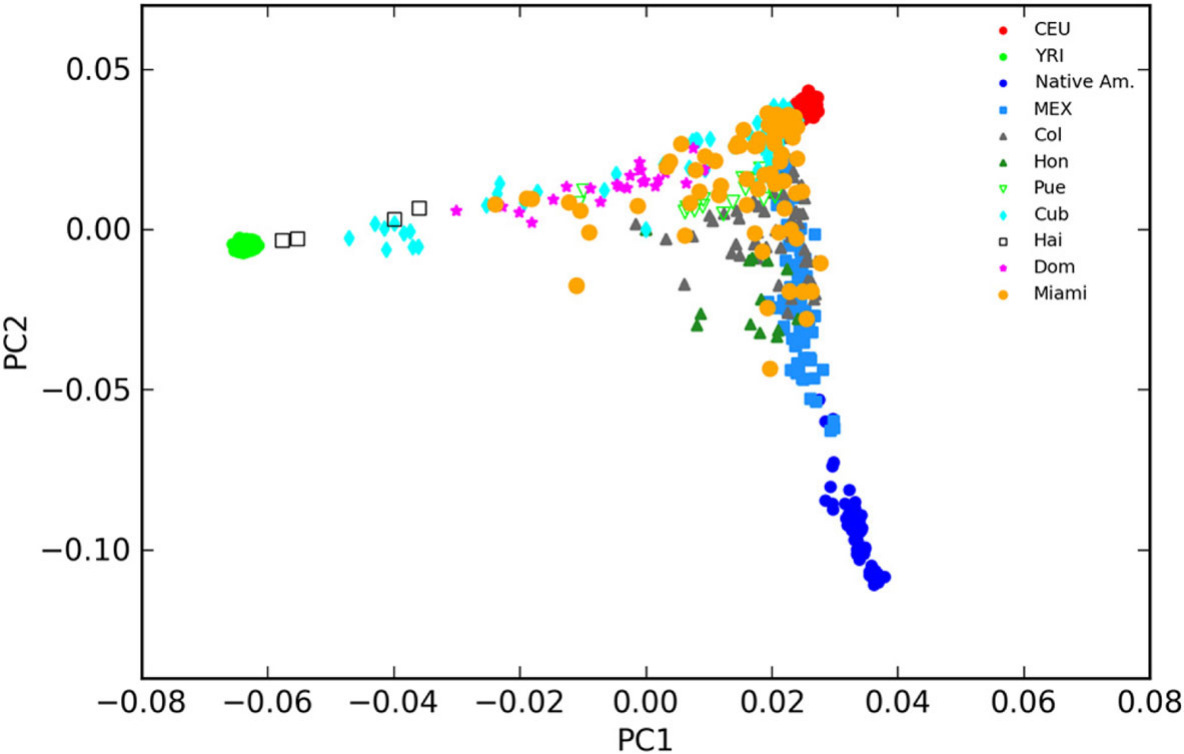
Plot of first (PC1) and second (PC2) principal component scores for each individual in the sample, indexed to the reference populations (GOAL and MEX samples). The components describe 66.2 and 26.6 % of the variation, respectively. PCs 3 and 4 capture variation among the Native American (HGDP) populations, but do not separate the study subjects. Includes Mexican (Mex), Colombian (Col), Honduran (Hon), Cuban (Cub), Puerto Rican (Pur), Haitian (Hai), Dominican (Dom), Central European (CEU), Yoruba African (YRI), and Native American (NativeAm) populations. Individuals from the present study are labeled “Miami”

**Fig. 5:**
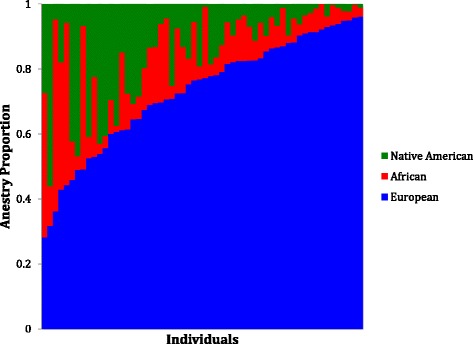
Plot of ancestry proportions for each sample ordered by European ancestry proportion (*European* CEU, *African* YRI, *Native American* NativeAm)

Figure 4 depicts the location of our 69 genotyped subjects along two principal component axes defined by ancestry-informative markers. Our samples tend to be more similar to the Central European population samples (median CEU ancestry of 0.77) than the African (YRI) or Native American (NatAm) samples, suggesting a predominantly European ancestry. There is sizeable overlap between our samples and the GOAL samples, representative of Hispanic populations throughout the Caribbean, South, and Central America (16). Figure 5 displays the ancestry proportion of each individual as a function of CEU, YRI, or NatAm contribution. There was no significant correlation between level of CEU ancestry and SRS-2 score (Pearson’s *r* = −.259, *p* > 0.05; Table 3), or between NatAm ancestry and SRS-2 score (Pearson’s *r* = 0.072, *p* > 0.5). There was a small positive correlation between YRI ancestry and SRS-2 score (Pearson’s *r* = 0.283, *p* = 0.03).

**Table 3:** Ancestry and SRS-2 correlations

	*n*	Pearson’s *r*	*p* value
CEU	57	−0.259	0.052
NatAm	57	0.072	0.595
YRI	57	0.283	0.033

There is a positive correlation between greater African (YRI) ancestry and higher SRS-2 score. There is no significant correlation between European (CEU) and Native American (NatAm) ancestry and SRS-2 score

## Discussion

This study confirmed substantial correlations in quantitative autistic trait (QAT) scores between first-degree relatives within and across generations, in a Hispanic cohort, and provides new validation data for such measurements in both English and Spanish versions of the assessment used, the Social Responsiveness Scale-2 (SRS-2). In an epidemiologic twin sample, we previously [18] showed that familial trait correlations indexed by the SRS-2 were not attributable to the effects of rater bias, and note that in this sample the correlations between unaffected children and their mothers (rated, respectively, by mothers and fathers) were as strong as the correlations between unaffected children and their fathers (in which case both ratings were made by mothers). The observation among ASD-affected families that the strength of association between children and their fathers was slightly higher than that between children and their mothers is fully in keeping with the results of prior ASD family studies [9, 26], which probably reflects a general reduction in the phenotypic expression of inherited liability for ASD among females in comparison to males [9, 27].

**Fig. 6:**
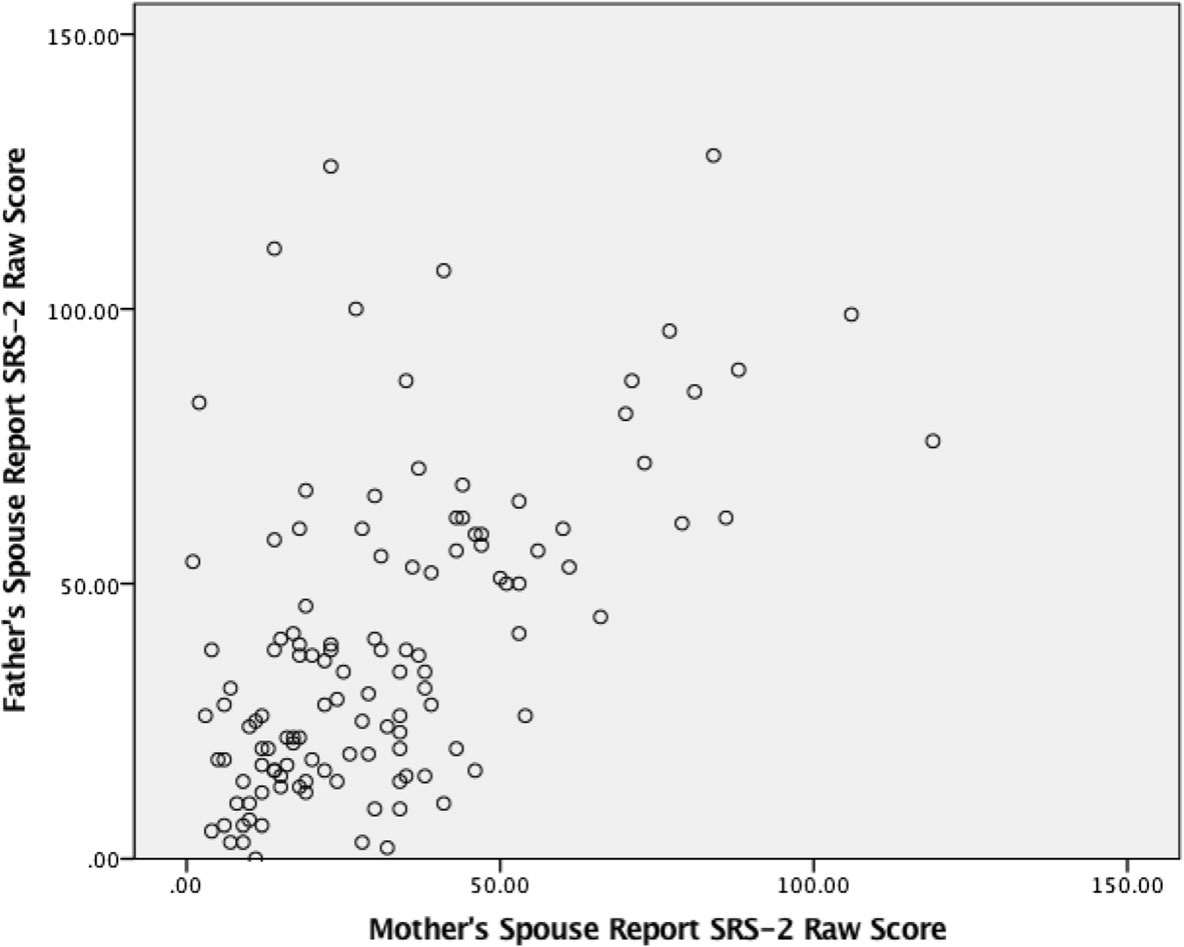
*Scatterplot* of spouse-report SRS-2 raw scores for the sample

Furthermore, the data obtained in this cohort provide cross-cultural evidence of pronounced effects of variation in social responsiveness on mate selection, and confirms prior observations that concordant elevation in QAT scores of parents is associated not only with elevated QAT scores of offspring but with increased offspring risk for *clinical*-ASD [9, 28]. Indeed, the spousal correlation of 0.48 in the ASD subgroup and 0.60 in the total sample are the largest spousal correlations observed to date for the SRS-2 data of couples; previous studies have reported spousal correlations of 0.38 [18], 0.26 [10], and 0.39 [9], among clinical and non-clinical Caucasian populations (Fig. 6).

In general, higher spousal correlations for an inherited trait operate to increase within-family aggregation of the traits over the course of successive generations [29]. Under assortative mating, the covariance of parent-offspring is given by CovPO = ½VA(1 + r) and for full siblings it is CovFS = ½VA(1 + rh^2^), where *r* is the phenotypic correlation for spouses and h^2^ the heritability of the trait. This generates the expectation that full siblings should be somewhat less similar to each other than to their parents, which is what was observed among ASD families in this sample [29].

An ICC value of 0.7 is the upper limit of what could be expected on the basis of inheritance alone for first-degree relations, and this would require 100 % inheritance of the trait. Therefore, the observation of very high trait correlations in this range suggests that despite the high heritability of SRS scores reported in family [8] and intergenerational [11] studies, other factors such as environmental and cultural influences may have contributed to ratings of first-degree relative concordance, as also suggested by the trend associated with ancestral origin. Of note is the possible influence of rater bias on ICC values was considered but relatively unlikely given prior studies demonstrating low rater bias effect for the SRS-2 [11] and the fact that we were able to observe similar concordance across different raters (i.e., mothers and fathers).

Furthermore, the observation that trait correlations between ASD probands and their first-degree relatives are *substantially* lower than those between unaffected first-degree relatives in ASD-affected families—which we have previously reported [9, 26]—is in keeping with recent molecular genetic studies which have suggested that ASD cases can arise from a *combination* of background genetic liability and superimposed de novo mutations, which exert pathological shifts against a genetic background [2, 5]. The effect of this in a mixed sample of families with and without such mutations contributing to clinical affectation in offspring would be to erode trait correlations between ASD probands and their first-degree relatives, due to the inclusion of probands with de novo mutations, in comparison to what is observed for other pairings of unaffected relatives within ASD-affected families. Another significant contributing factor to the erosion of the correlation between ASD probands and their relatives is the effect of clinical ascertainment—affected individuals possess a greater burden of rare inherited variants than their unaffected relatives [30]. The result of this “oversampling” of risk alleles will be to lower the trait correlation between affected individuals and their relatives [31, 32].

Evidence in other species shows that assortative mating over the span of several generations can have a rapid and significant evolutionary impact on the demographics of a population [33]. The association between concordant elevation of subclinical traits in spousal pairs and clinical affectation in their offspring—is one of several manifestations of the impact of additive genetic factors on the development of autism. Furthermore, *sub clinical* elevation in quantitative autistic trait burden may be evolutionarily *adaptive* in certain contexts, though not in the extreme [34]. For example, traits such as focus-on-detail or heightened object orientation might be advantageous for food gathering in primates or technical job performance in the modern era. To the extent that genetic variation might confer such advantages to males while preserving rearing behavior in females may have implications for the universal and pronounced sex ratio observed in ASD, a highly inherited condition for which the majority of molecular genetic variants are autosomal (not sex-linked). There are other examples of continuously distributed traits (such as height) in which incremental increases confer higher relative fitness, the adaptive value of which sharply decreases at the extreme end of distribution [35]. More generally, in balancing selection, extreme ends of the spectrum are less favorable, while heterogeneity tends to be preserved in the population [36].

Individuals of Hispanic identity are exceptionally diverse in ancestry and genetic origin compared to many other populations [23], and our genotypic analysis of 69 Hispanic children with ASD confirmed the ancestral diversity of our sample. Although predominantly of European descent, there was a large admixture of Native American and African ancestral origin as well. Moreno-Estrada et al., [23] noted that Hispanic individuals with more Native American ancestry are more likely to come from Mexico, Central, or South America, while individuals with more African ancestry are more likely to originate from the Caribbean islands. Our heterogeneous population sample was expected considering the unique demographics of South Florida Hispanic communities. A limitation of this study was the relatively modest sample size of individuals genotyped, which severely limited statistical power to draw inferences from associations between phenotype and broad ancestral analysis [28].

Finally, the Spanish language test yielded somewhat higher average SRS-2 scores for children with ASD and their parents. The complex history of Hispanic ancestry and colonialism is a potentially important factor when relating ancestry to biological traits and may contribute to correlations between language and genetic ancestry [23]. The complex interplay between ancestry and SRS-2 scores is further demonstrated by the small correlation between Yoruban ancestry and elevated SRS-2 scores. The association is difficult to interpret in that it may be influenced by cultural differences in interpretation of the SRS-2, as well as by health and social disparities that broadly correlate with ancestral origin in the USA. Specifically, socio-economic status [37–39] is directly correlated with ascertainment rates of ASD diagnosis. When fewer cases are diagnosed in a population, the diagnosed cases of ASD tend to be more severe [39]; this and inverse associations between SES and language/executive functioning [40] could have contributed to higher mean SRS-2 scores seen in ASD-affected families who completed the Spanish language version of the test.

## Conclusions

The present study provides strong evidence of familial aggregation of quantitative autistic traits (QAT) between generations (from parents to offspring) and within generations (between siblings) among Hispanic families. These traits represent measurable indices of inherited liability to ASD that may relate to common genetic variants. We also observed strong evidence of assortative mating for social behavioral characteristics indexed by the Social Responsiveness Scale, and this reflects a mechanism by which ASD traits might accumulate within families in successive generations. The degree to which this occurs may vary across cultures, and measurement of QATs in prospective parents may represent important indices of risk for the occurrence of ASD in their offspring.

## Abbreviations

ADI-R: Autism Diagnostic Interview-Revised
ASD: Autism spectrum disorder
CEU: Population of central Europeans in Utah
CHARGE: Childhood autism risk from genetics and environment
GOAL: Genetic admixtures and origins of latinos study
QAT: Quantitative autistic traits
SCQ: Social communication questionnaire
SRS-2: Social responsiveness scale-2
YRI: Population of Yoruba in Africa
